# Retinal Layer Abnormalities as Biomarkers of Schizophrenia

**DOI:** 10.1093/schbul/sbx130

**Published:** 2017-12-19

**Authors:** Niraj N Samani, Frank A Proudlock, Vasantha Siram, Chathurie Suraweera, Claire Hutchinson, Christopher P Nelson, Mohammed Al-Uzri, Irene Gottlob

**Affiliations:** 1 University of Leicester Medical School, Leicester, UK; 2 Ulverscroft Eye Unit, University of Leicester, Leicester, UK; 3 Department of Neuroscience, Psychology and Behaviour, University of Leicester, Robert Kilpatrick Clinical Sciences Building, Leicester Royal Infirmary, UK; 4 Leicestershire Partnership NHS Trust, Bradgate Unit, Glenfield Hospital, Leicester, UK; 5 NIHR Leicester Cardiovascular Biomedical Research Unit, Glenfield Hospital, Leicester, UK; 6 Adult Social and Epidemiological Psychiatry and Disability Research Group, Department of Health Sciences, University of Leicester, Leicester General Hospital, Leicester, UK

**Keywords:** schizophrenia, biomarkers, optical coherence tomography (OCT), contrast sensitivity, retinal layers

## Abstract

**Objective:**

Schizophrenia is associated with several brain deficits, as well as visual processing deficits, but clinically useful biomarkers are elusive. We hypothesized that retinal layer changes, noninvasively visualized using spectral-domain optical coherence tomography (SD-OCT), may represent a possible “window” to these abnormalities.

**Methods:**

A Leica EnvisuTM SD-OCT device was used to obtain high-resolution central foveal B-scans in both eyes of 35 patients with schizophrenia and 50 demographically matched controls. Manual retinal layer segmentation was performed to acquire individual and combined layer thickness measurements in 3 macular regions. Contrast sensitivity was measured at 3 spatial frequencies in a subgroup of each cohort. Differences were compared using adjusted linear models and significantly different layer measures in patients underwent Spearman Rank correlations with contrast sensitivity, quantified symptoms severity, disease duration, and antipsychotic medication dose.

**Results:**

Total retinal and photoreceptor complex thickness was reduced in all regions in patients (*P* < .0001). Segmentation revealed consistent thinning of the outer nuclear layer (*P* < .001) and inner segment layer (*P* < .05), as well as a pattern of parafoveal ganglion cell changes. Low spatial frequency contrast sensitivity was reduced in patients (*P* = .002) and correlated with temporal parafoveal ganglion cell complex thinning (*R* = .48, *P* = .01). Negative symptom severity was inversely correlated with foveal photoreceptor complex thickness (*R* = −.54, *P* = .001) and outer nuclear layer thickness (*R* = −.47, *P* = .005).

**Conclusions:**

Our novel findings demonstrate considerable retinal layer abnormalities in schizophrenia that are related to clinical features and visual function. With time, SD-OCT could provide easily-measurable biomarkers to facilitate clinical assessment and further our understanding of the disease.

## Introduction

Schizophrenia is a chronic debilitating psychiatric condition that ranks among the leading causes of global disease-related disability^1^. Its clinical presentation is wide-ranging and reliable investigations are generally lacking. The discovery of biomarkers of schizophrenia is highly desirable but equally challenging due to our limited understanding of its pathological basis. The classical theory of dopamine excess has been broadened to include a newer model of glutamatergic dysfunction that may contribute to negative symptoms.^2^ Postmortem experiments of the schizophrenic brain have shown gray matter structural deficits, including dendritic spine density reductions in prefrontal cortical regions.^3^ This has been supported by MRI studies demonstrating gray matter volume loss in >48 regions that show various associations with disease features.^4^ However, MRI has not yet yielded clinically applicable biomarkers and is a time-consuming, noisy test that may be impractical for paranoid patients.

**Fig. 1.**
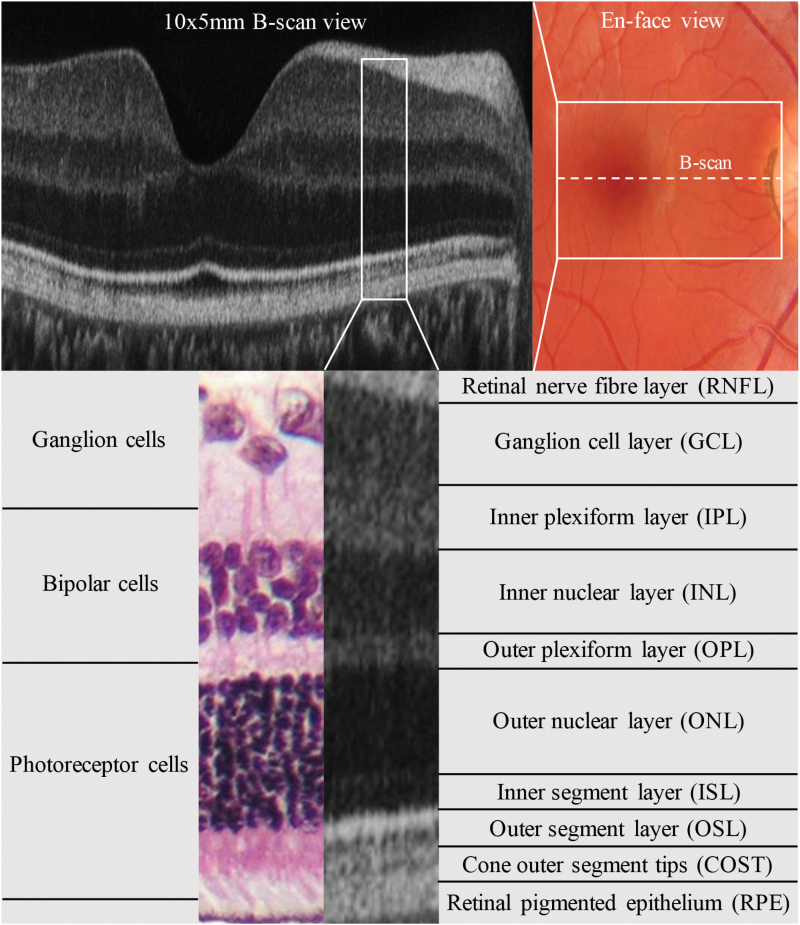
SD-OCT of the macula. SD-OCT 10 mm × 5 mm B-scan of the foveal center (top left) and en-face view of the macula with a corresponding 10 mm × 5 mm scan box and cross-sectional line through the central fovea indicating the location of the B-scan (top right). A labeled breakdown of the individual retinal layers (bottom) in the SD-OCT nasal parafoveal region (right middle) with a histological correlate (left middle).

Spectral-domain optical coherence tomography (SD-OCT) is a fast noninvasive imaging technique that provides in vivo visualization of the retinal layers,^5^ particularly at the macula and its foveal center (figure 1). This region contains a high density of ganglion cells that are specialized to differentiate contrast, with magnocellular and parvocellular subtypes conferring low and high spatial frequency vision, respectively.^6^ Ganglion cell axons converge to form the peripapillary retinal nerve fiber layer (pRNFL) and, in turn, the optic nerve. The retina is considered to be a directly observable “window” to the brain.^7^ Retinal neurons originate from forebrain neuroectoderm and lack myelin, with distinct layers containing components of gray matter such as cell bodies and dendrites as well as various neurotransmitters. SD-OCT has been increasingly employed to search for quantifiable biomarkers of neurological and psychiatric diseases. pRNFL thinning and macular volume deficits have been consistently reported in multiple sclerosis, the latter being correlated with disability and MRI deficits.^8^ However, comparable findings in Alzheimer’s and Parkinson’s disease suggest these measures are nonspecific.^9,10^ Advances in SD-OCT scan resolution and analysis now allow thicknesses of all individual retinal layers to be measured using image segmentation. Distinct layer changes have already been demonstrated in multiple sclerosis^11^ and the accuracy of automated segmentation techniques continues to improve.^12^ Early findings of SD-OCT investigations looking for schizophrenia biomarkers have been controversial. Studies have reported reduced pRNFL thickness^13–15^ and macular volume,^15^ one finding a significant correlation with disease duration with both measures.^15^ However, other investigations have only observed isolated deficits^16,17^ or none at all.^18^ The most recent study found global RNFL thinning, as well as ganglion cell layer (GCL) and inner plexiform layer (IPL) volume deficits.^19^ To the best of our knowledge, no previous studies have performed macular segmentation of all individual retinal layers in schizophrenia.

There is a growing body of evidence for early-stage visual processing impairments in schizophrenia, but contrast sensitivity research is a particularly contentious area. Several studies have reported greater contrast sensitivity reductions with lower spatial frequency stimuli,^20–22^ which has been attributed to a magnocellular pathway deficit through evidence from visual backward masking^23^ and electrophysiological investigations.^24,25^ This has been implicated in reading^26^ and facial emotion recognition^27^ impairments in schizophrenia. However, low spatial frequency contrast sensitivity reductions have not been reported in other investigations^22^ and have yet to be ascribed to structural changes in the retina or brain.^28^ This has led to criticism of the magnocellular deficit theory.^29^

We hypothesize that brain abnormalities in schizophrenia can manifest as specific retinal layer changes that may be associated with disease features and visual impairment. We conducted a case–control study comparing individual retinal layer thickness measurements at 3 macular regions, obtained through manual segmentation of high-resolution SD-OCT images, in patients with schizophrenia and demographically matched controls. We also compared contrast sensitivity measurements in a subgroup of each cohort. In patients with schizophrenia, we correlated abnormal layer parameters with contrast sensitivity, symptom severity, disease duration, and antipsychotic drug dose.

## Materials and Methods

### Participant Recruitment

Thirty-five patients with schizophrenia and 50 controls were recruited into the study at Glenfield Hospital, Leicester, United Kingdom between November 2014 and June 2015. This sample was based on a priori power calculation of SD-OCT data from 72 healthy adults showing that 34 subjects in each group would be required to detect a 5% difference in inner nuclear layer (INL) thickness (mean difference = 1.79 µm, standard deviation = 2.24 µm, power = 90%, α = 0.05). The study received ethical approval from the Leicestershire Research Ethics Committee and was conducted in accordance with the Declaration of Helsinki. Participant exclusion was assessed through a structured questionnaire and reviewed by senior clinicians. Exclusion criteria included any previously diagnosed ophthalmological diseases, diabetes mellitus, ocular surgery/trauma, optical spherical equivalent of ≥+6 or ≤−6 dioptres and substance dependency. All patients met International Classification of Diseases, Tenth edition (ICD-10) F20 diagnostic criteria for schizophrenia^30^ and were recruited consecutively from the community (*n* = 18), ward (*n* = 13), and clinic (*n* = 4). Controls without any diagnosed psychiatric condition were recruited from hospital staff (*n* = 47) and unaffected spouses of cases (*n* = 3).

### Disease Characteristics

Symptom severity in patients was quantified using the Positive and Negative Syndrome Scale (PANSS), a rating tool of 7 positive (PANSS-P), 7 negative (PANSS-N), and 16 general (PANSS-G) symptoms of schizophrenia.^31^ This was achieved through a structured clinical interview conducted within a week of SD-OCT examination by a trained psychiatrist. Disease duration was extrapolated as years from the date of first official diagnosis. Equivalent daily dose of antipsychotic medication was calculated as a percentage of the maximum daily dose as defined by the British National Formulary (https://www.bnf.org/).

### SD-OCT Assessment

The Leica Envisu hand-held SD-OCT system (Bioptigen) was employed to acquire retinal scans in both eyes of participants. The hand-held probe was mounted and subjects were positioned on a chin-head rest to focus upon an external fixation target. A 5-second volumetric 10 mm × 5 mm scan of the foveal center, marked by outer segment layer (OSL) thickening, was captured. Each scan consisted of 500 A-scans/B-scan, 50 B-scans and 5 frames/B-scan, with acceptable scans containing ≥5 consecutive B-scan frames of the foveal center with no movement artefacts. An “averaging” protocol was implemented in ImageJ (http://imagej.nih.gov/ij/) to isolate, align and combine frames to generate smoothed B-scan images of the foveal center. Manual segmentation of the individual retinal layers was performed using an ImageJ macro and this process was performed masked by allocating random numbers to B-scan images before analysis. Average individual and combined layer thickness measurements were extracted from 3 macular regions relative to the foveal center (0 µm): (1) foveal region = −750 to 750 µm, (2) nasal parafoveal region = −1500 to −750 µm and (3) temporal parafoveal region = 750 to 1500 µm. Individual layer thickness measurements included: (1) retinal nerve fiber layer (RNFL), (2) ganglion cell layer (GCL), (3) inner plexiform layer (IPL), (4) inner nuclear layer (INL), (5) outer plexiform layer (OPL), (6) outer nuclear layer (ONL), (7) inner segment layer (ISL), (8) outer segment layer (OSL), (9) cone outer segment tips (COST), and (10) retinal pigmented epithelium (RPE). Combined thickness measurements included: (1) total retina = RNFL − RPE inclusive, (2) photoreceptor complex = ONL − COST inclusive, (3) processing complex = RNFL − OPL inclusive, and (4) ganglion cell complex = GCL + IPL.

### Visual Acuity and Contrast Sensitivity Assessment

Best-corrected monocular visual acuity was assessed using the Freiburg Acuity Test^32^ in both eyes of participants. Only eyes with visual acuity of logMAR ≤0.2 (Snellen ≤ 20/32) were included for contrast sensitivity testing. If both eyes achieved this threshold, the right eye was selected at random. Contrast sensitivity was measured in this selected eye in a subgroup of 24 patients with schizophrenia and 44 controls. Contrast detection thresholds were measured at 0.5, 2, and 8 cycles per degree (cpd) spatial frequencies for stationary sinusoidal gratings that subtended 6 degrees in horizontal and vertical dimensions. Gratings were spatially windowed according to a half-cycle of a raised cosine function to prevent spatial transients. Stimuli were generated in C programming language using a Macintosh G4 and presented on a Sony CRT monitor with an update rate of 75 Hz. For precise control of luminance contrast the number of intensity levels available was increased to 14 bits using a Bits++ attenuator (Cambridge Research Systems). The monitor was gamma-corrected using a spot photometer (LS-100, Konica Minolta) and look-up-tables. A single-interval forced-choice psychophysical procedure was employed. On each trial participants were presented with a fixation cross, followed by a stimulus. Participants had to judge the stimulus pattern orientation (vertical or horizontal), which was randomized on each trial. Grating contrast varied according to a modified 1-up 3-down staircase designed to converge on the luminance contrast corresponding to 79.4% correct performance.^33^ At the beginning of each trial-run the contrast was set to 6 dB above threshold. On subsequent reversals the step-size was halved and testing was terminated after 16 reversals. Threshold estimates were taken as the mean of the last 4 reversals in each staircase. Contrast thresholds were converted to contrast sensitivity (1/threshold).

### Statistical Analysis

SD-OCT measurements were assessed for normality and analyzed for group differences using a linear mixed model in Stata (StataCorp, College Station, TX). The model compared residuals calculated from a linear regression adjusted for eye (right/left), age, gender and ethnicity. Contrast sensitivity measurements were log-transformed and analyzed for group differences using a linear model adjusting for age, gender, and ethnicity. Additional analyses also adjusting for spherical equivalent were performed for each comparison. SD-OCT measurements that exhibited statistically significant differences between patients and controls underwent further analysis for associations with contrast sensitivity measurements, PANSS scores, disease duration, and antipsychotic drug dose with bivariate Spearman Rank correlations using residuals from a linear model adjusted for age, gender, and ethnicity.

## Results

### Participant Characteristics

The characteristics of patients with schizophrenia and controls included in the comparisons are shown in table 1. Patient and control groups were well-matched for age, gender, and ethnicity. Visual acuity was significantly poorer in patients [logMAR = 0.07 (±0.21)] compared with controls [logMAR = −0.06 (±0.18)] in the SD-OCT comparison (*P* = .01). Spherical equivalent was significantly lower in patients [−1.4 (±2.0) dioptres] compared with controls [−0.1 (±1.7) dioptres] in the contrast sensitivity comparison (*P* = .01). However, spherical equivalent and visual acuity were not measured in 8 patients due to glasses being forgotten/lost.

**Table 1.** Group Characteristics

	SD-OCT Comparison			Contrast Sensitivity Comparison		
Characteristics	*Patients*	*Controls*	*P*	*Patients*	*Controls*	*P*
Overall (*N*)	35	50	—	24	44	—
Gender (*N*)			1.00			
Male	25	35		16	28	1.00
Female	10	15		8	16	
Ethnicity (*N*)			1.00			.49
Caucasian	25	34		17	32	
Indian	6	10		3	9	
Afro-Caribbean	3	4		3	2	
Other	1	2		1	1	
Age (years)	40.6 (12.9)	40.6 (12.7)	.95	36.0 (9.0)	39.4 (12.7)	.95
Visual acuity (logMAR)	0.07 (0.21)	−0.06 (0.18)	.01	0.00 (0.19)	−0.08 (0.14)	.06
Spherical equivalent (dioptres)	−0.8 (2.1)	−0.6 (2.2)	.31	−1.4 (2.0)	−0.1 (1.7)	.01
Disease duration (years)	16.3 (9.1)	—	—	15.4 (7.6)	—	—
PANSS score (arb. units)		—	—		—	—
PANSS-P	15.5 (6.5)			15.1 (6.3)		
PANSS-N	15.2 (7.6)			14.8 (7.1)		
PANSS-G	25.6 (7.1)			25.9 (7.2)		
Antipsychotic drug		—	—		—	—
Typical (*N*)	6			2		
Atypical (*N*)	29			22		
% Maximum daily dose	59.2 (41.3)			55.7 (43.1)		
CPZE (mg)	387.9 (273.5)			380.8 (293.4)		

*Note*: Cohort details of patients with schizophrenia and control subjects participating in the main SD-OCT comparison (left) and secondary contrast sensitivity comparison (right) with mean (and standard deviation) values. LogMAR, logarithm of the minimum angle of resolution (0.2 logMAR = 20/32, 0 logMAR = 20/20, −0.2 LogMAR = 20/12.5); PANSS, Positive and Negative Syndrome Scale; PANSS-P, positive symptom severity; PANSS-N, negative symptom severity; PANSS-G, general symptom severity; CPZE, chlorpromazine equivalent (dose equivalent to 100 mg chlorpromazine). Group *P*-values were acquired in continuous data using a *t*-test and in categorical data using Fisher’s exact test.

### SD-OCT Comparison

**Fig. 2.**
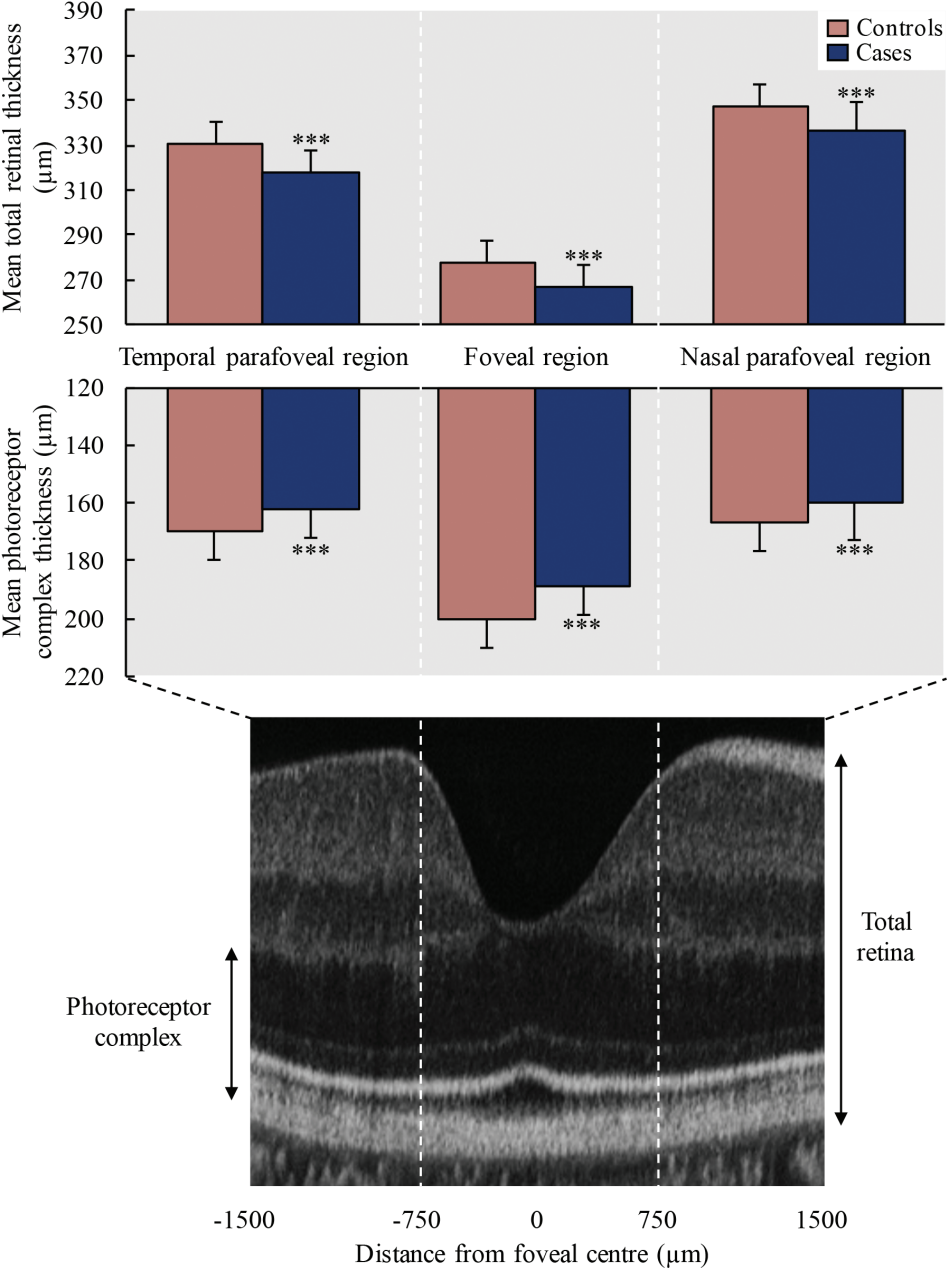
SD-OCT retinal complex thickness comparison. Clustered column charts comparing mean total retinal thickness (top) and mean photoreceptor complex thickness (bottom) between patients with schizophrenia and controls in temporal parafoveal (left), foveal (middle), and nasal parafoveal (right) regions. A corresponding SD-OCT central foveal B-scan image is included below to identify these regions. *P ≤ .05, **P ≤ .01, ***P ≤ .001.

**Fig. 3.**
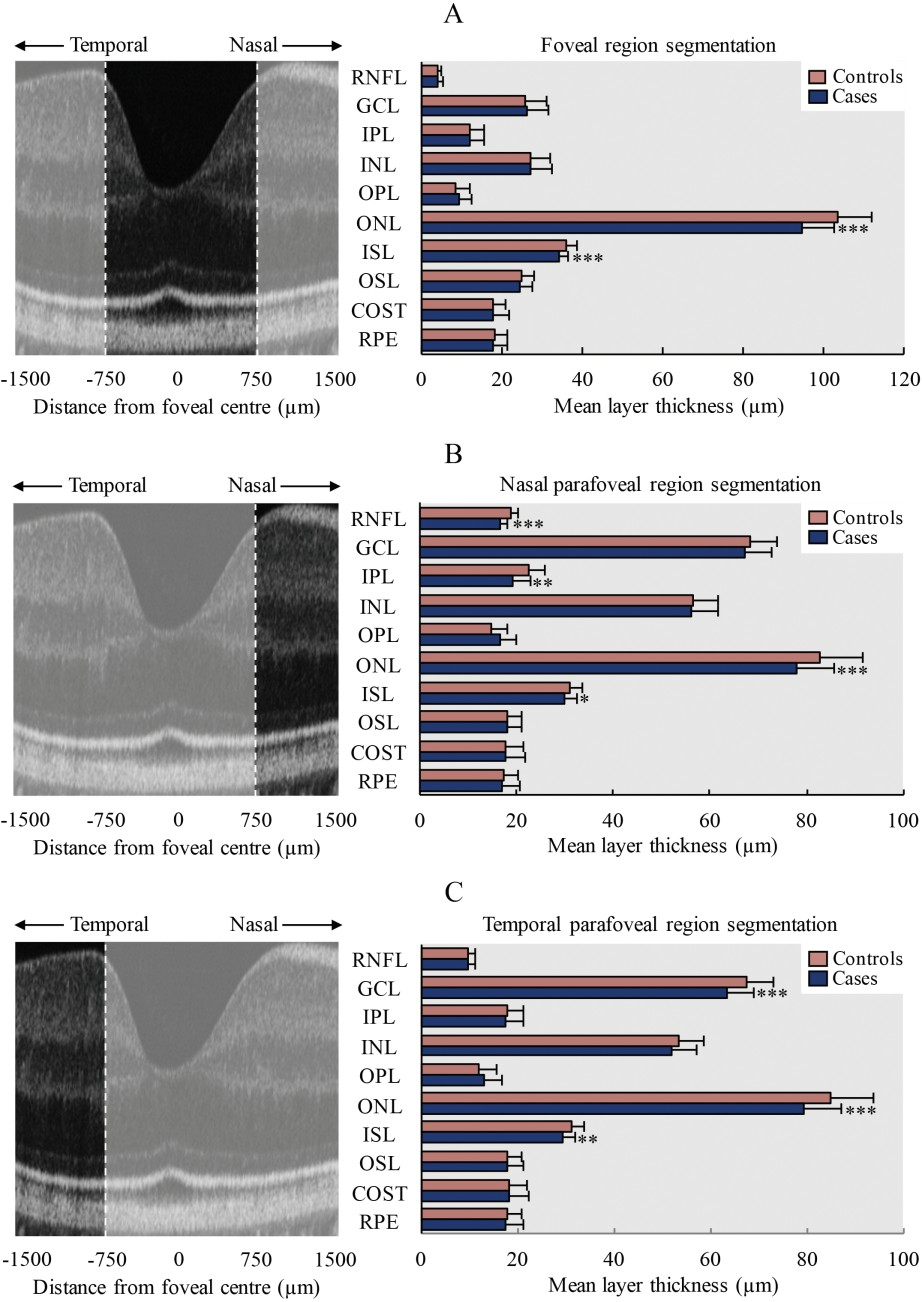
SD-OCT retinal layer segmentation comparison. Bar charts comparing mean and standard deviation of layer thickness measurements (right) between patients with schizophrenia and controls and corresponding SD-OCT central foveal B-scan images (left) to outline the region analyzed in darker shade (A) foveal region, (B) nasal parafoveal region, and (C) temporal parafoveal region. RNFL, retinal nerve fiber layer; GCL, ganglion cell layer; IPL, inner plexiform layer; INL, inner nuclear layer; OPL, outer plexiform layer; ONL, outer nuclear layer; ISL, inner segment layer; OSL, outer segment layer; COST, cone outer segment tips; RPE, retinal pigmented epithelium. *P ≤ .05, **P ≤ .01, ***P ≤ .001.

Total retinal thickness was significantly reduced in the foveal region [β = −18.5 µm (±6.1 µm), *P* < .0001], nasal parafoveal region [β = −12.2 µm (±5.3 µm), *P* < .0001] and temporal parafoveal region [β = −15.4 µm (±5.3 µm), *P* < .0001] in patients compared with controls. This was largely due to photoreceptor complex thickness being significantly reduced in the foveal region [β = −13.8 µm (±3.8 µm), *P* < .0001], nasal parafoveal region [β = −9.2 µm (±4.5 µm), *P* < .0001] and temporal parafoveal region [β = −10.8 µm (±3.9 µm), *P* < .0001] (figure 2). Individual layer segmentation results are summarized in figure 3. In patients, ONL thickness was significantly reduced in the foveal region [β = −9.4 µm (±3.1 µm), *P* < .0001], nasal parafoveal region [β = −6.9 µm (±4.0 µm), *P* = .0007], and temporal parafoveal region [β = −6.9 µm (±3.0 µm), *P* < .0001]. ISL thickness was also significantly reduced in the foveal region [β = −2.0 µm (±0.9 µm), *P* < .0001], nasal parafoveal region [β = −1.2 µm (±1.0 µm), *P* = .02] and temporal parafoveal region [β = −1.6 µm (±1.1 µm), *P* = .007]. GCL thickness was significantly reduced in the temporal parafoveal region [β = −3.5 µm (±1.1 µm), *P* = .001], whereas in the nasal parafoveal region there were significant reductions in RNFL thickness [β = −2.5 µm (±1.0 µm), *P* < .0001], and IPL thickness [β = −2.8 µm (±2.1 µm), *P* = .004]. Ganglion cell complex thickness was significantly reduced in the temporal parafoveal region [β = −3.9 µm (±2.9 µm), *P* = .009]. An additional adjustment for spherical equivalent did not affect the significance of any results.

### SD-OCT Correlations With Disease Outcomes

**Fig. 4.**
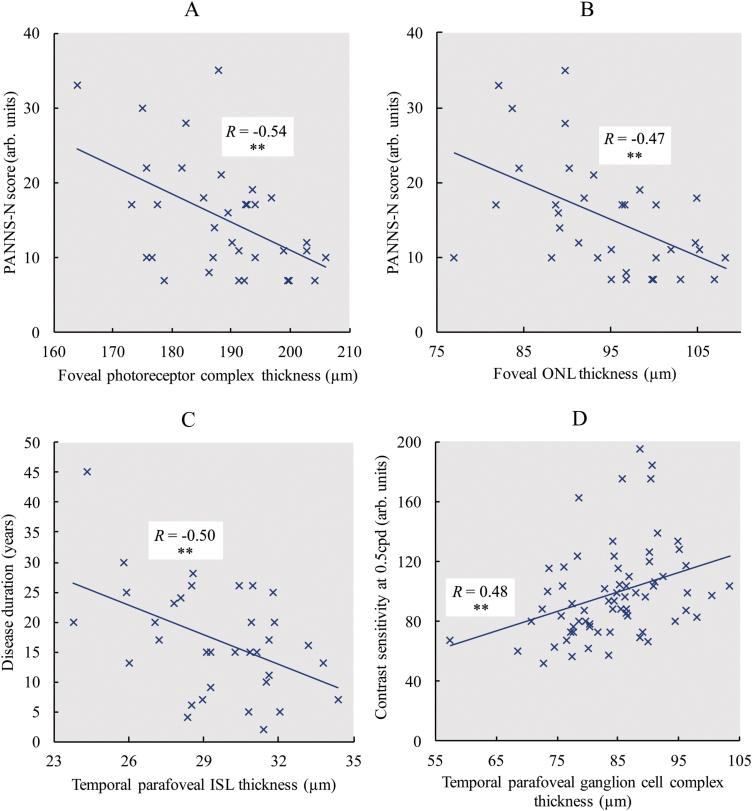
Correlations between SD-OCT and clinical parameters in cases. Scatter graphs in patients with schizophrenia (marked by X) showing Spearman Rank correlations (with corresponding adjusted *R*-values and significance levels) between (A) foveal photoreceptor complex thickness and PANSS-N (negative symptom severity) score, (B) foveal outer nuclear layer (ONL) thickness and PANSS-N score, (C) temporal parafoveal inner segment layer (ISL) thickness and disease duration, and (D) temporal parafoveal ganglion cell complex thickness and contrast sensitivity at 0.5 cpd spatial frequency. *P ≤ .05, **P ≤ .01, ***P ≤ .001.

PANSS-N score (negative symptom severity) displayed significant inverse correlations with foveal photoreceptor complex thickness (*R* = −.54, *P* = .001) (figure 4A) and foveal ONL thickness (*R* = −.47, *P* = .005) (figure 4B) in patients. Significant inverse correlations with PANSS-N score were also observed for nasal parafoveal total retinal thickness (*R* = −.35, *P* = .04) and nasal parafoveal photoreceptor complex thickness (*R* = −.38, *P* = .03). PANSS-G score (general symptom severity) demonstrated a significant inverse correlation with nasal parafoveal ONL thickness (*R* = −.37, *P* = .03). Disease duration showed significant inverse correlations with temporal parafoveal ISL thickness (*R* = −.50, *P* = .003) (figure 4C), temporal parafoveal ONL thickness (*R* = −.46, *P* = .006), and temporal parafoveal photoreceptor complex thickness (*R* = −.41, *P* = .02). No significant correlations were observed between antipsychotic drug dose and any abnormal SD-OCT measure.

### Contrast Sensitivity Comparison and Correlations

Contrast sensitivity was significantly reduced at 0.5 cpd spatial frequency in patients compared with controls [β = −0.77 (±0.48), *P* = .002]. The significance of this result did not change when also adjusting for spherical equivalent (*P* = .01). In patients, 0.5 cpd contrast sensitivity displayed significant correlations with temporal parafoveal total retinal thickness (*R* = .61, *P* = .002) and temporal parafoveal ganglion cell complex thickness (*R* = .48, *P* = .01) (figure 4D). Correlations with this measure were also significant for foveal total retinal thickness (*R* = .42, *P* = .04) and foveal photoreceptor complex thickness (*R* = .42, *P* = .04). These correlations were not replicated in controls.

## Discussion

Our study identified retinal layer changes in schizophrenia using SD-OCT. Photoreceptor complex thinning, particularly of the ONL and ISL, was uniform and exhibited a distinct association with negative symptom severity. A pattern of inner layer changes was also observed in parafoveal regions and temporal parafoveal ganglion cell complex thinning was correlated with a selective low spatial frequency contrast sensitivity deficit.

We observed macular thinning, which is consistent with recent comparably-sized investigations and denotes retinal neuronal loss.^15,16^ In addition, Lee et al^15^ detected an inverse correlation between macular thickness and disease duration that implies a neurodegenerative process supported by evidence of progressive gray matter volume loss from MRI studies.^34^ Our study only found this association with temporal parafoveal photoreceptor measures, which is a subtle deficit that cannot be positively attributed to global neurodegeneration. Ascaso et al^16^ reported no correlation with disease duration but instead found macular thinning that was limited to subjects with nonrecent illness episodes. This prompts an alternative suggestion that the changes are state-dependent rather than progressive but this has not been explored in our investigation. Yilmaz et al^17^ found only isolated macular thinning in nasal and inferior outer regions. However, unlike our study, groups were not matched for ethnicity and this may represent a possible confounding factor. Several prior studies have not observed any macular deficits in schizophrenia,^13,14,18^ most notably a large investigation conducted by Chu et al.^18^ However, this study may have been confounded by inclusion of cases with schizoaffective disorder whilst our recruitment was based upon strict ICD-10 diagnostic criteria. In comparison to all previous studies, ours was well-powered and used an SD-OCT device with superior axial resolution (2.4 µm). This, in addition to using a novel image “averaging” method, facilitated accurate segmentation of individual retinal layers for the first time in schizophrenia.

Our study isolated a structural photoreceptor deficit, with ONL and ISL thinning being consistent and highly significant. One previous study reported ONL thinning in multiple sclerosis that was associated with faster disability progression, but unlike our study INL thinning was also found.^11^ We observed that foveal photoreceptor and ONL thinning correlated with negative symptom severity, assessed using the well-validated PANSS tool by a single trained assessor.^35^ NMDA glutamatergic receptor hypoactivity has been implicated in the pathogenesis of negative symptoms^36^ and the same receptors also mediate glutamatergic cone photoreceptor bipolar cell pathways at the fovea.^37^ Therefore, photoreceptor thinning may represent a manifestation of NMDA dysfunction. It must be noted that NMDA receptor hypofunction has also been linked with positive symptoms for which correlations were not observed. ISL thinning may be linked, but this layer is particularly mitochondria-rich and thus our result may reflect well-evidenced mitochondrial abnormalities in schizophrenia.^38,39^ Our finding of parafoveal ganglion cell deficits is consistent with previous studies reporting pRNFL thinning,^13–15^ as well as global GCL and IPL volume reductions.^19^ However, the specific distribution of nasal RNFL and IPL thinning but temporal GCL thinning was unexpected and requires replication in independent samples. Ganglion cell loss may result from reduced glutamatergic input secondary to photoreceptor NMDA receptor dysfunction,^40^ or abnormal modulatory dopamine-mediated connections with amacrine cells.^41^ Mutations in schizophrenia susceptibility genes expressed in the inner retina may also play a role. Neuregulin-1 promotes survival of retinal ganglion cells^42^ whereas dysbindin is localized to Müller glial cells that support neuronal growth^43^ and contribute to the electroretinogram b-wave,^44^ in which deficits have been observed in schizophrenia.^45^ The retinal layer changes detected by our study may help reconcile previous findings to focus biomarker research. This may provide new insights into the neurobiological processes underlying schizophrenia and SD-OCT could eventually become an adjunct to routine clinical assessment.

We also detected a distinct low spatial frequency contrast sensitivity reduction. This is consistent with several previous investigations^20–22^ and corresponds to a specific magnocellular pathway deficit in schizophrenia. The observed correlation with temporal parafoveal ganglion cell complex thinning may signify disease-related magnocellular ganglion cell loss. This provides to a possible structural basis to magnocellular dysfunction in schizophrenia that has been shown to impact on real-world functioning.^28^ However, other studies have failed to replicate a similar deficit^22^ and this may result from heterogeneity in methodological factors such as luminance level.^29^ The impact of reduced visual acuity, despite being adjusted for, and cognitive factors in the schizophrenia cohort also cannot be discounted.^29^

We recognize several limitations to our investigation. Only 30% of approached suitable subjects with schizophrenia engaged in our study. This may reflect the nature of the disease but raises the possibility of ascertainment bias that may restrict the generalizability of our findings. Similarly, mean PANSS scores showed that symptoms were rated between 2 (minimal) and 3 (mild) on average and this restricted range may have limited our ability to detect correlations. Spherical equivalent and visual acuity measurements were not obtained from 8 recruited patients with schizophrenia due to glasses being forgotten or lost. This group may somehow differ from the remaining cohort that is not being accounted for. We employed a manual segmentation technique to assess individual layers with greater accuracy than current automated systems, but this method is more time-consuming and open to human error. However, our segmentation method was blinded and showed high test/re-test reliability in a previous study using the same device.^46^ Our study performed multiple statistical comparisons, which may warrant a post hoc correction. For a significance threshold of *P* ≤.05, the Bonferroni level for 14 layer comparisons is *P* = .0036 and the majority of layer differences remain significant despite this correction. However, the comparisons were not independent as layer and combined measurements are inherently correlated between and within regions and therefore such a correction was not adopted. SD-OCT changes showed no correlation with current antipsychotic drug dose in our study, but this did not account for historical doses or polypharmacy. Given the dopaminergic innervation to the retina and known effects of neuroleptic drugs on visual function,^28^ it is difficult to disregard the possible influence of these medications without testing a drug-naïve sample.

In conclusion, our study demonstrated retinal layer abnormalities in schizophrenia that were related to negative symptoms and contrast sensitivity. These novel findings warrant future investigations in this developing subfield of retinal biomarker research in neuropsychiatry.

## Funding

N.N.S. was supported by a Wolfson Foundation award for intercalating students. F.A.P. and I.G. are supported by the Ulverscroft Foundation and the Medical Research Council (MR/J004189/1 and MRC/N004566/1).
